# Phosphocreatine Improves Cardiac Dysfunction by Normalizing Mitochondrial Respiratory Function through JAK2/STAT3 Signaling Pathway *In Vivo* and *In Vitro*

**DOI:** 10.1155/2019/6521218

**Published:** 2019-11-30

**Authors:** Eskandar Qaed, Jiaqi Wang, Marwan Almoiliqy, Yanlin Song, Wu Liu, Peng Chu, Sawsan Alademi, Maria Alademi, Hailong Li, Mohammed Alshwmi, Mahmoud Al-Azab, Anil Ahsan, Samar Mahdi, Guozhu Han, Mengyue Niu, Amr Ali, Abdullah Shopit, Hongyan Wang, Xiaodong Li, Abdullah Qaid, Xiaodong Ma, Tong Li, Jinyong Peng, Jing Ma, Jianbin Zhang, Zeyao Tang

**Affiliations:** ^1^Department of Pharmacology, Dalian Medical University, Dalian, Liaoning 116044, China; ^2^Department of Plastic and Reconstructive Surgery, The First Hospital of Jilin University, 1500 Qinghua Road, Changchun 130021, China; ^3^College of Medical Sciences, Taiz University, Yemen; ^4^Department of Immunology Guangzhou Institute Pediatrics, Guangzhou Woman and Childrens Medical Center, Guangzhou Medical University, Guangzhou, 510623, China; ^5^N.I. Pirogov Russian National Research Medical University, Russia

## Abstract

Diabetic cardiomyopathy (DCM) is one of the common cardiovascular complications in patients with diabetes. Accumulating evidence has demonstrated that DCM is thoroughly related to mitochondrial energy impairment and increases the generation of reactive oxygen species (ROS). Therefore, an ongoing study is developing strategies to protect cardiac mitochondria from diabetic complications, especially from hyperglycemia. Phosphocreatine (PCr) plays a major metabolic role in cardiac muscular cells including intracellular concentration of ATP which affects the activity of the myocardium. We hypothesized that PCr might improve oxidative phosphorylation and electron transport capacity in mitochondria impaired by hyperglycemia in vivo and in vitro. Also, we aimed to evaluate the protective effect of PCr against DCM through the JAK2/STAT3 signaling pathway. The mitochondrial respiratory capacity from rats and H9C2 cells was measured by high-resolution respirometry (HRR). Expressions of proteins Bax, Bcl-2, caspase 3, caspase 9, cleaved caspase 3, and cleaved caspase 9, as well as JAK2/STAT3 signaling pathways, were determined by western blotting. ROS generation and mitochondrial membrane potential (MMP) were measured with fluorescent probes. Type 1 diabetes mellitus was induced in Wistar male rats by a single intraperitoneal injection of streptozotocin (STZ) (80 mg/kg body weight). Our results revealed that PCr possessed protective effects against DCM injury by improving the mitochondrial bioenergetics and by positively exerting protective effects against DCM in vivo and in vitro, not only improving diabetes symptom, resulting in changes of cardiac tissue using hematoxylin and eosin (H&E) stain, but also ameliorating biochemical changes. Moreover, PCr increased Bcl-2, caspase 3, and caspase 9 protein expressions and decreased Bax, cleaved caspase 3, and cleaved caspase 9 expressions as well as the JAK2/STAT3 signaling pathway. In conclusion, PCr improves mitochondrial functions and exerts an antiapoptotic effect in vivo and in vitro exposed to oxidative stress by hyperglycemia through the JAK2/STAT3 signaling pathway. Our findings suggest that PCr medication is a possible therapeutic strategy for cardioprotection.

## 1. Introduction

Recent studies have demonstrated that diabetic cardiomyopathy (DCM) is the main sequence of diabetes mellitus (DM). DCM is characterized with inconsistent increase in left ventricular (LV) muscle [1–4]. Moreover, recent researches have showed that mitochondrial energy metabolism variation is one of the common causes of heart disease including DCM [5]. In addition, mitochondria are essential and important regulators of cellular bioenergetics to provide the normal heart its daily need of ATP. In fact, about 40% of the cytoplasmic space in adult cardiac myocyte is employed by mitochondria. Mitochondrial oxidative phosphorylation (OXPHOS) in the respiratory chain (RC) complexes locates in their inner membrane occupying the majority of high demand for ATP [6]. Therefore, myocardial mitochondria are highly responsive to any injury through high energy demand substrate accessibility which plays a very important role in heart stability. On the other hand, clinical studies have indicated that mitochondrial dysfunction contributes to cardiomyopathy with several clinical indicators. Moreover, mitochondrial dysfunction and diminished energy creation have been detected in various formulas of heart illnesses including DCM [7]. Likewise, the revelation of a novel helpful procedure for the advancement and upkeep of a mitochondrial work is of extraordinary logical significance in the treatment of DCM [8]. High generation of reactive oxygen species (ROS) is the main cause of progression to cardiac brokenness when mitochondrial vitality is impaired [9]. Recently, oxidative stress has been recognized as a risk factor in the progressing of diabetic cardiovascular complications [10]. Oxidative stress caused by higher production or reduced degradation of ROS is involved crucially in physiological and pathological processes of cell life and death decisions [11, 12], for example, apoptosis. It has been accounted for that apoptosis of cardiomyocytes is one of the fundamental outcomes of hyperglycemia-actuated oxidative stress in the myocardium [13]. Cardiomyocyte apoptosis in diabetic creature models and patients is expanded because of the loss of contractile tissues, rebuilding, and at last brokenness [14, 15]. It has been known that cardiomyocyte apoptosis is related to a few pathways like extrinsic pathway induced by ligands fixed to death receptors and the intrinsic pathway managed by the arrival of a few genius apoptotic proteins from the mitochondria [16].

Phosphocreatine (PCr) is a high vitality phosphate compound which goes as a vitality provider and has the double capacity of stacking and dispatching ATP in vitality digestion [17]. Though exogenous PCr offers vitality straightforwardly to the cell through the creatine transport, different investigations have suggested that PCr is essential for supporting the vitality digestion of apoptotic cells which is through keeping up picture dependability [18]. Currently, PCr can be synthesized artificially. Exogenous PCr has recently been used as a cardioprotective drug due to its superior efficacy in the protection of the myocardium against hypoxia injury and its significant improvement [19]. PCr has been added to the cardioplegia arrangement and conveyed before the start of a cardiopulmonary detour by means of intravenously [20]. The Janus kinase/signal transducer and activator of transcription (JAK/STAT) signaling pathway is an intracellular pathway which accepts a key part in cell improvement, survival, and directing quality articulation [21, 22]. Upon phosphorylation by JAK/STAT, proteins translocate into the nucleus to muddle to the sponsor area of target genes and control their transcription. In the heart, STATs adjust the appearance of genes encoding proteins concerned in development, extracellular matrix composition, inflammation, apoptosis, and cellular signaling. In addition, JAK/STAT plays a serious role in the expansion of heart failure and cardiac hypertrophy [23]. Inhibition of JAK2 may offer a novel helpful methodology in the treatment of diabetic inconvenience in the vasculature of the STZ-actuated diabetic rats. JAK2 phosphorylation is a basic stage in the enhancement of diabetic vascular complexities [24]. Hyperglycemia is a key clinical indication of diabetes mellitus which has been found to expand the age of ROS [25].

Based on the above considerations, we proposed a speculation that PCr might have the defensive impact of diabetic cardiomyocytes against a dangerous effect of hyperglycemia by the regulation of heart mitochondrial respirometric states through the JAK2/STAT3 signaling pathway. Moreover, we supposed the therapeutic activity of PCr through reducing ROS subsequently decreasing apoptosis. What we are concerned with our test drug, PCr, could protect against DCM and whether it is related to the mitochondrial respiratory chain and signal conduction pathway. In any case, the issues of an inclusion of the JAK2/STAT3 pathway tweak in the defensive impacts of PCr against DCM have not been accounted yet after we have reviewed the cardioprotective effect of PCr in vivo and in vitro hyperglycemia-prompted concentration on its impact on the JAK2/STAT3 signaling pathway.

## 2. Materials and Methods

### 2.1. *In Vivo* Experimental Design

#### 2.1.1. Animal

In the present experiment, forty Wistar male rats (4~6 weeks old and 150~200 g) were used. The approval to conduct this study was granted by the ethical committee of human and animal research of the Dalian Medical University. The study was conducted in accordance with Guide for the Care and Use of Laboratory Animals [26]. The rats were kept in steady situations at room temperature (21~23°C) with a 12/12 light/dark cycle. After three weeks of adaptation, the rats were divided into four groups: (1) healthy rats, (2) diabetic rats, (3) diabetic rats injected with low-dose PCr (20 mM), and (4) diabetic rats injected with high-dose PCr (50 mM).

#### 2.1.2. Induction of Diabetes

STZ is a glucosamine-nitrosourea compound that shows particular cytotoxicity to pancreatic cells. It is utilized to actuate trial creature diabetes. Type 1 diabetes was instigated in Wistar male rats (4~6 weeks old and 150~200 g body/weight). The rats were given an intraperitoneal freshly prepared solution of STZ (80 mg/kg body weight) dissolved in sodium citrate buffer (0.1 M, pH 4.2; Sigma-Aldrich, USA). The rats were housed in a steadied domain kept up in the research center with a consistent temperature from the start of our examination. Accordingly, rats were divided into the following groups: (1) control—nondiabetic rats simply accepting water, (2) STZ—diabetic rats that have been presented to STZ and given fresh water, (3) treatment-PCr—diabetic rats accepting low-dose PCr (20 mM) intravenously once daily over the span of the investigation, and (4) treatment-PCr—diabetic rats accepting high-dose PCr (50 mM) intravenously once daily for 12-week treatment over the span of the investigation. The measurement of fasting blood glucose was measured using a blood glucose meter (OneTouch UltraEasy, Edina, MN, USA) (centralization higher than 16.7 mmol/L).

#### 2.1.3. Blood Glucose and Cardiac Marker Measurement

Fasting blood glucose (stately dependably at 08:00~9:00 AM) was estimated once in nondiabetic rats and watched weekly in all STZ group as well as treatment with the PCr group. We utilized exceptional needle prick to collect the blood from tail vessels. The last assurance of blood glucose was recorded utilizing glucose strips at a time going before the basic event (weekly) and alluded correspondingly to terminal glucose. Moreover, the myocardial catalyst markers such as malondialdehyde (MDA), superoxide dismutase (SOD), and glutathione (GSH) from tissues were estimated as indicated by the manufacturer's instructions (Nanjing Jiancheng Bioengineering Institute, China).

#### 2.1.4. Tissue Accumulation and Histology

At the end of the analysis, the hearts of the rats were extracted and weighed, and we have taken a photograph of the heart size to evaluate the difference that happened in the estimation; after that, left ventricles were fixed in 4% supported paraformaldehyde and paraffin and separated. In each group, not less than 5 arbitrarily chosen areas were recolored with hematoxylin and eosin (H&E) according to the manufacturer's instructions and photographed using a light microscope (Nikon Eclipse TE2000-U, NIKON, Japan).

#### 2.1.5. Immunofluorescence Staining

Tissue area slides were settled with 4% paraformaldehyde for 20 min at room temperature and after that flushed with PBS (phosphate-buffered saline) for 5 min and incubated in a permeabilization with 0.4% TritonX-100 for 10 min. The slides were washed with PBS three times for 5 min each time and then blocked with 15% bovine serum albumin (BSA) for 30 min in PBS, then washed three times with PBS and incubated with a p-STAT3 counteracting agent at 4°C overnight; after being washed with PBS three times for 10 min, fluorescein-conjugated secondary antibody was added in 1% solution of blocking and incubated for 1 h each. Subsequently, cell nuclei were stained with DAPI (1 *μ*g/mL for 10 min). The samples were examined using a fluorescence microscope (CKX4, OLYMPUS, Japan).

#### 2.1.6. TUNEL Assay

The measurement of in vivo apoptotic cell death was performed using TUNEL assay as indicated according to the manufacturer's instructions (Roche, Germany). DAPI was incorporated in the unit, as DNA pieces could be stained by TUNEL particularly and delivered green fluorescence. Quickly, tissue sections were settled with 4% paraformaldehyde at room temperature and afterwards were washed with PBS for 5 min and hatched in permeabilization with 0.4% TritonX-100 for 10 min and washed with PBS three times. The TUNEL response blend was included, and the samples were incubated with CO_2_ at 37°C for 1 h. The sections were stained with 1 *μ*g/mL DAPI for 10 min. The apoptotic rate was demonstrated by TUNEL-positive cell number against the aggregate cell number with DAPI under a fluorescence microscope (CKX4, OLYMPUS, Japan).

#### 2.1.7. High-Resolution Respirometry

**Figure 1:**
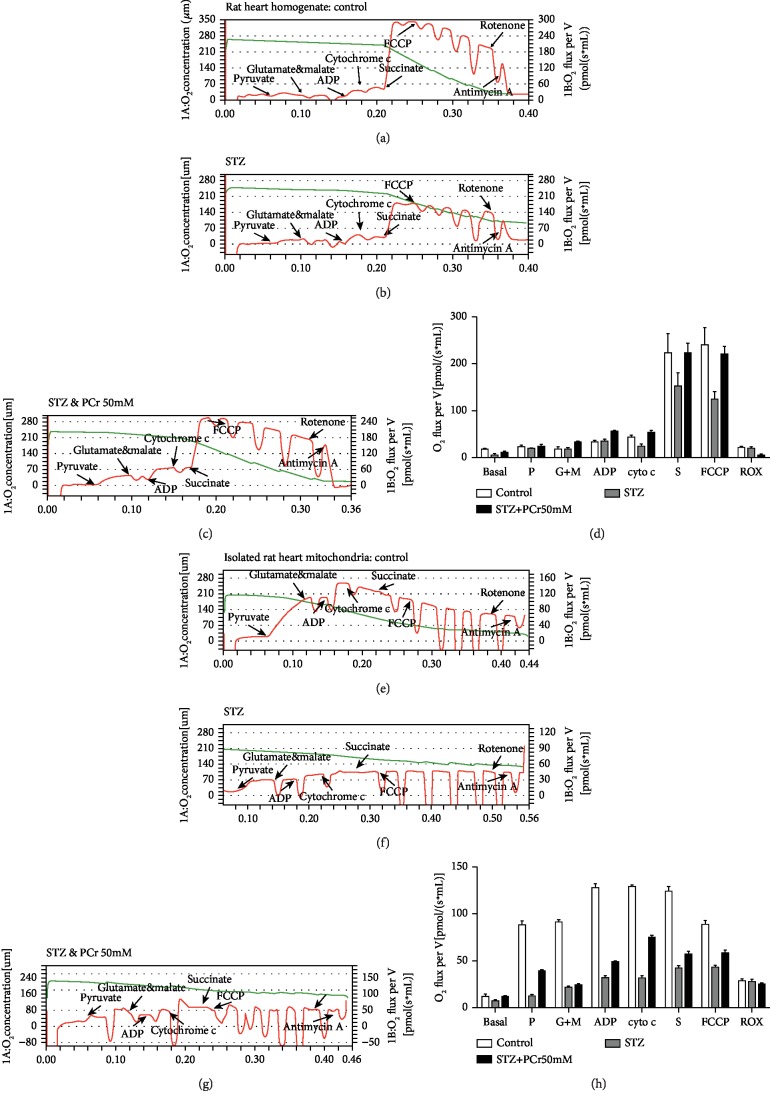
Mitochondrial respiration in heart tissue homogenate and isolated heart mitochondria of rats. (a) Healthy heart rat tissue homogenate and the mitochondrial respiration were detected using Oxygraph-2k. Red lines indicate the oxygen consumption rate in response to sequential loading of mitochondrial effectors (indicated by arrows above the graphs). (b) STZ group. (c) The treatment group with PCr (50 mM). (d) Quantitative examinations of oxygen utilization rate in light of effectors. Correlations were performed utilizing data lab programming. (e) Isolated heart mitochondria from healthy rats. (f) STZ group. (g) The treatment group with PCr (50 mM). (h) Quantitative examinations of oxygen utilization rate in light of effectors. The following are added: P: pyruvate (5 mM); G: glutamate (10 mM); M: malate (2 mM); ADP: adenosine diphosphate (5 mM); Cyt C: cytochrome c (10 *μ*M); S: succinate (10 mM); FCCP (0.5 *μ*M steps); rotenone (0.5 *μ*M); antimycin A (2.5 *μ*M). Data are displayed as the mean ± SE (*n* = 3 per group), and comparisons were performed using DatLab software.

Oxygen consumption was estimated by high-resolution respirometry utilizing Oxygraph 2k (Oroboros Instruments GmbH, Innsbruck, Austria) according to the manufacturer's instructions [27]. All substrates and inhibitors were included as portrayed in Figure 1. Investigations utilizing heart tissue homogenate and isolated heart mitochondria were performed in MiR05 (110 mM sucrose, 60 mM K-lactobionate, 0.5 mM EGTA, 3 mM (MgCl_2_), 20 mM taurine, 10 mM (KH_2_PO_4_), 20 mM (HEPES), and 1 g/L BSA pH 7.1). Data were dissected using Oroboros DatLab 5.1 software. O2k instruments (two chambers) were used. All the investigations were performed at 37°C.

#### 2.1.8. Isolation of Cardiac Mitochondria

Rats were anesthetised by intraperitoneal infusion of thiopental (0.1 g/kg). Heart tissue for each group was homogenized gently, and heart mitochondria were separated from the treated group and diabetes rats individually. Mitochondria were isolated according to the standard protocol [28]. With a little alteration, the left ventricle was quickly removed from euthanized rats [2.5 mg/g wet weight (heart)]. Then it was immediately placed in small volume of ice-cold isolation solution (containing 250 mM sucrose, 2 mM EDTA, 10 mM Tris, and 1 g/L BSA, pH 7.4) and was cut into small pieces with scissors and left together with 10 mL of isolation solution with the addition of dispase II (Sigma-Aldrich, D 4693). Then, the pieces of the heart were transferred to a teflon/glass homogenizer and homogenized gently for 2~3 min. After centrifugation of the homogenized sample at 800× g for 10 min at 4°C, the protease containing supernatant with a part of mitochondria which were in a direct contact with the protease was centrifuged at 4800× g for 10 min, at 4°C. Then, the pellet was resuspended in the same volume of isolation solution, but without protease, and was again homogenized and spun down at 4800× g for 10 min at 4°C. The last centrifugation of the pellet was done at the same conditions as described above. Finally, the pellet containing mitochondria was again resuspended in the ice-cold isolation solution (buffer, pH 7.4). Mitochondrial protein content was determined by the bicinchoninic acid (BCA) (Bio-Rad, Hercules, CA, USA). The respiration of isolated mitochondria from rat heart and heart tissue homogenate were determined using substrate-uncoupler inhibitor titration (SUIT) protocols with modifications, then transferred to mitochondrial respiration medium (MiR05) [0.5 mM EGTA, 3 mM MgCl_2_, 60 mM K-lactobionate, 20 mM taurine, 10 mM KH_2_PO_4_, 20 mM HEPES, 110 mM D-sucrose, and 1 g/L BSA (Sigma-Aldrich; A3803) changed in accordance with pH 7.1]. The following are used: pyruvate (P) (5 mM), glutamate (G) (10 mM), and malate (M) (2 mM), which were first added as substrates for mitochondrial respiration; adenosine diphosphate (ADP) (5 mM) was added to induce state 3 respiration; Cyt C (cytochrome c) (10 *μ*M) was added to test the integrity of the outer mitochondrial membrane; succinate (S) (10 mM) was added for electron transfer to complex II; then FCCP (0.5 *μ*M steps), a mitochondrial respiration uncoupler, was added to obtain maximal oxygen consumption rate; rotenone (0.5 *μ*M) was added to inhibit complex I; antimycin A (2.5 *μ*M) to inhibit complex III was added for the determination of residual oxygen consumption (ROX).

#### 2.1.9. Protein Extraction and Western Blot Analysis

Heart tissue homogenate was lysed. The proteins were separated on (10~ 15%) sodium dodecyl sulfate-polyacrylamide gel electrophoresis (SDS-PAGE) and then electrically transferred onto a polyvinylidene difluoride (PVDF) membrane. The protein concentrations of the samples were determined by BCA. The membranes were visualized using enhanced chemiluminescence reagent with LabWorks software (UVP, Upland, CA, USA). The analogous experiments were performed at least three times.

### 2.2. *In Vitro* Experimental Design

#### 2.2.1. Cell Culture and Treatment

H9C2 cardiomyoblast was bought from the Organization of Natural Chemistry Cell Biology (Shanghai, China). Phosphocreatine (PCr) was bought from Harbin Laiboten Pharmaceutical Co., Ltd. Methylglyoxal (MGO), 3-(4,5-dimethylthiazol-2-yl)-2,5-diphenyltetrazolium bromide (MTT), streptomycin, and penicillin were obtained from Sigma-Aldrich (St. Louis, MO, USA). The MDA, SOD, and GSH kits were acquired from Nanjing Jiancheng Bioengineering Institute (Nanjing, China). 5,58,6,68-Tetraethyl benzimidazol carbocyanine iodide (JC-1) were purchased from Fanbo Biochemicals. 2′,7′-Dichlorodihydrofluorescein diacetate (DCFH-DA) was acquired from Beyotime (Jiangsu, China). The antibodies such as JAK2, STAT3, anti-phospho-STAT3, and anti-phospho-JAK2 were obtained from Bioworld Technology (USA). Anti-Bax, anti-Bcl-2, anti-caspase 3, anti-caspase 9, cytochrome c (mitochondria), lamin B1, and *β*-actin were obtained from Proteintech Group, Inc. (Chicago, IL, USA). In the present study, we utilized Dulbecco's modified Eagle medium (DMEM) which contains 10% fetal bovine serum (FBS), 100 U/mL of penicillin, and 100 U/mL of streptomycin. H9C2 cells were cultured and treated with different concentrations (5~40 mM) of PCr and then incubated with CO_2_ at 37°C. PCr-pretreated cells were gradually stimulated with MGO (0.2~1.2 mM). Furthermore, a solution of different concentrations of PCr was diluted with DMEM to give a final concentration of 20 mM. PCr concentration was subjected to a test to identify the toxic effect. Also, the final concentration of MGO was 1 mM. For high-resolution respirometry and O2k-Fluorometry, all the materials such as pyruvate, glutamate, malate, succinate, cytochrome c, rotenone, oligomycin, FCCP, digitonin, antimycin A, and ADP were obtained from Sigma-Aldrich. All the reagents and solvents used in this study were of the highest analytical reagent grade.

#### 2.2.2. MTT Assay

The H9C2 cells were plated in 96-well plates for 24 h at a density of 1 × 10^6^ cells/mL and treated with different concentrations of PCr (5~20 mM) and N-acetyl cysteine (NAC) (2 mM) for 2 h, then stimulated for 24 h with MGO (1 mM). The reasonability of cells was evaluated by the MTT method. Additionally, for morphological appearance examination, the cells were plated in 6-well plates and pretreated with or without PCr (5~20 mM) and NAC (2 mM) for 2 h, individually, then stimulated with MGO (1 mM) for 24 h. The images were obtained by using an inverted microscope (Nikon, Japan).

#### 2.2.3. DAPI Staining

The H9C2 cells (1 × 10^6^ cells/mL) were seeded in 6-well plates and incubated at 37°C for 24 h. Then, the cells were treated with PCr (5~20 mM) or NAC (2 mM) concentrations of test compounds for 2 h, then stimulated with MGO (1 mM) for 24 h. Once the incubation time was done, the cells were washed two times with PBS (phosphate-buffered saline). The cells were stained with DAPI (1 *μ*g/mL) diluted in asepsis water. The pictures were evaluated by a fluorescence microscope (OLYMPUS, Japan).

#### 2.2.4. Detection of Cell Apoptosis

After the indicated treatments, the treated H9C2 cells were harvested, washed three times with ice-cold PBS, and assessed for apoptosis using an Annexin V-fluorescein isothiocyanate (FITC) and propidium iodide (PI) double staining kit according to the manufacturer's instructions (Nanjing KeyGen Biotech. Co. Ltd., Nanjing, China). The percentages of apoptotic cells were investigated by flow cytometry (Becton Dickinson, USA).

#### 2.2.5. Detection of Mitochondrial Membrane Potential

The H9C2 cells (1 × 10^6^ cells/mL) were seeded overnight in 6-well plates and pretreated for 2 h with and without PCr (5~20 mM) individually, then stimulated with MGO (1 mM) for 24 h. The cells were flushed with the DMEM, incubated with JC-1 (10 *μ*g/mL) for 15 min at 37°C. After that, cells were washed two times with PBS. The samples were measured using a fluorescence microscope (CKX4, OLYMPUS, Japan).

#### 2.2.6. Determination of Cellular Respiration

Oxygen consumption was estimated by high-resolution respirometry utilizing Oxygraph 2k (Oroboros Instruments GmbH, Innsbruck, Austria) according to the manufacturer's instructions. The H9C2 cells (1 × 10^6^ cells/mL) were seeded overnight in 6-well plates and pretreated for 2 h with and without PCr (20 mM) individually, then stimulated with MGO (1 mM) for 24 h; after the incubation time, the cells were harvested and loaded with MiR05 in addition to 1 mg/mL cells suspended in 2.2 mL warm MiR05 and moved to chambers in the O2K. The following are used: digitonin (8.1 *μ*M, 10 *μ*g/10^6^ cells) to permeabilize the plasma membranes completely only affecting mitochondrial membranes at higher concentrations, pyruvate (P) (5 mM), glutamate (G) (10 mM), and malate (M) (2 mM) which were first added as substrates for mitochondrial respiration; adenosine diphosphate (ADP) (5 mM) which was added to induce state 3 respiration; succinate (S) (10 mM) which was added for electron transfer to complex II; FCCP (0.5 *μ*M steps), a mitochondrial respiration uncoupler, which was added to obtain maximal oxygen consumption rate; rotenone (0.5 *μ*M) which was added to inhibit complex I; and antimycin A (2.5 *μ*M) to inhibit complex III added for the determination of residual oxygen consumption (ROX). Additionally, the intact H9C2 cells (1 × 10^6^ cells/mL) were seeded overnight in 6-well plates and pretreated with and without PCr 20 mM individually for 2 h, then stimulated with MGO (1 mM) for 24 h. The cells were harvested and loaded with DMEM, then moved to chambers in the O2K. The following are used: after adjustment of ROUTINE respiration, the ATP-synthase inhibitor oligomycin (2.5 *μ*M) added to get a measure of LEAK respiration, then FCCP (0.5 *μ*M steps); rotenone (0.5 *μ*M); and antimycin A (2.5 *μ*M) added for the determination of residual oxygen consumption (ROX). All the investigations were performed at 37°C.

#### 2.2.7. Detection of Intracellular ROS Production

The H9C2 cells (1 × 10^6^ cells/mL) were seeded overnight in 6-well plates and pretreated with and without PCr (20 mM) individually for 2 h, then stimulated with MGO (1 mM) for 24 h. The cells were gathered and after that stacked with 500 *μ*L of DCFH diacetate (10 mM) at 37°C for 20 min, subsequent to washing twice with PBS. The samples were investigated by flow cytometry (Becton Dickinson, USA).

#### 2.2.8. Immunofluorescence Staining

The effect of PCr on the nuclear translocation of p-STAT3 was examined by immunofluorescence staining. For the immunofluorescence staining of p-STAT3, the formalin-fixed H9C2 cells were incubated with anti-p-STAT3 antibodies overnight at 4°C. Then, after being washed with PBS three times for 10 min, the fluorescein-conjugated secondary antibody was added in 1% blocking solutions and incubated for 1 h. Subsequently, cell nuclei were stained with DAPI (1 *μ*g/mL for 10 min). The samples were examined using a fluorescence microscope (CKX4, OLYMPUS, Japan).

#### 2.2.9. Protein Extraction and Western Blot Analysis

The H9C2 cells (1 × 10^6^ cells/ml) were cultured in 6-well plates and pretreated with PCr (5~20 mM) for 2 h, respectively, then stimulated with MGO (1 mM) for 24 h. Total cytosolic proteins were extracted with a cold lysis buffer (100 *μ*M PMSF) for 10 min on ice; then the mixtures were centrifuged at 12000 × g for 15 min at 4°C, and the supernatant was collected. Proteins were separated using SDS-PAGE and then electrically transferred onto a polyvinylidene difluoride (PVDF) membrane. The protein concentrations of the samples were determined by BCA. The membranes were visualized using enhanced chemiluminescence reagent with LabWorks software (UVP, Upland, CA, USA). The analogous experiments were performed at least three times.

### 2.3. Statistical Analysis

Data were analyzed using GraphPad Prism 5 (Graph Pad Software, Inc., San Diego, CA) and expressed as the mean and standard deviation. Statistical evaluations of post hoc multiple group comparisons were conducted using one-way ANOVA. A Bonferroni test was used for statistical analysis. *P* value < 0.05 was considered statistically significant.

## 3. Results

### 3.1. *In Vivo* Experiments

#### 3.1.1. PCr Lightens Histopathologic Changes in the Myocardium of DCM

**Figure 2:**
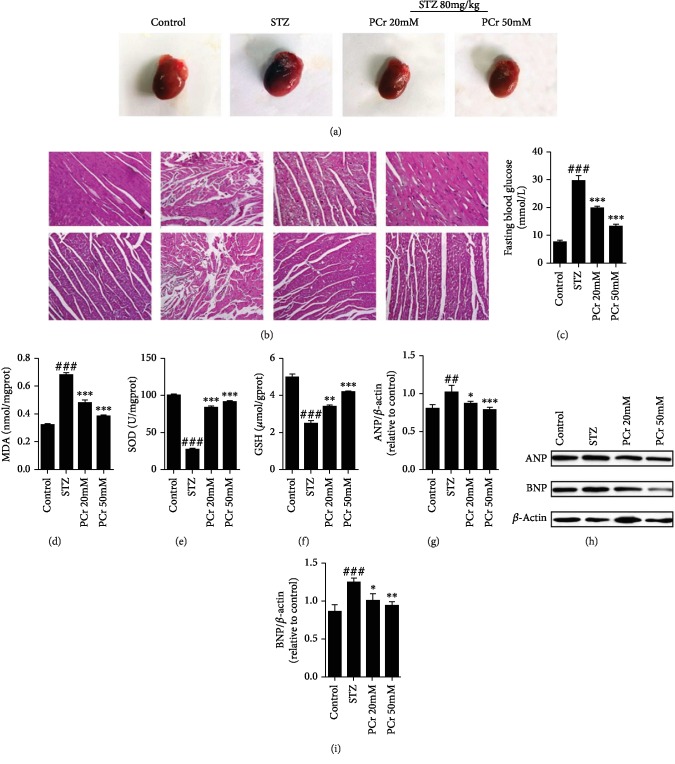
PCr decreased pathological changes in diabetic hearts. (a) Effects of PCr on heart size were reduced significantly in treated groups than the diabetic group. (b) Hematoxylin and eosin (H&E) staining of the heart tissue showed that PCr improved the striation of myocardial fibers arranged regularly in treated groups. (c) Effects of PCr on blood glucose levels were reduced remarkably in the treated groups than the STZ group. (d) Effects of PCr on myocardial and antioxidative enzyme activities: MDA level was decreased significantly in the PCr group than in the diabetic group; (e, f) SOD and GSH were increased in treated groups compared with diabetic groups indicating the antioxidant capacity agent was endangered in the diabetic myocardium. (g, h, i) Western blot investigation of atrial natriuretic peptide (ANP) and brain natriuretic peptide (BNP). Data are introduced as the mean ± SD (*n* = 3). ^###^*P* < 0.01*vs.* control; ^∗∗^*P* < 0.01 and ^∗∗∗^*P* < 0.01*vs.* the STZ group.

Heart weight was higher in diabetic rats than control and treated rats as shown in (Figure 2(a)). Also, we confirmed our result using western blot for ANP and BNP as shown in (Figures 2(g)–2(i)). Additionally, H&E staining as shown in (Figure 2(b)) was performed to illuminate the impact of PCr on the histopathologic changes in the myocardium. It was resolved that the cardiomyocytes were obviously striated and routinely showed in the control rats, while confused and central rot cells were exhibited in the STZ rats. This was enhanced after treatment with the low and high portion of PCr. Surely, the histopathologic changes in the high portion PCr assemble enhanced to a more noteworthy degree than the low portion in the treatment gathered with PCr, demonstrating that PCr offers a defensive impact against the DCM.

#### 3.1.2. Effects of PCr on Blood Glucose

Blood glucose, water charge, and sustenance utilization body weight (72 h after STZ infusion) of the rats extraordinarily expanded, and the rats additionally showed traditional side effects of diabetes, including expanded water charge and nourishment utilization and polyuria. High glucose builds the osmotic weight of the pee because of expanded liquid misfortune, causing drying out and expanded thirst; the body cannot make full utilization of glucose because of insulin inadequacy, which prompts absence of vitality and results in polyphagia for the whole investigation. The outcomes demonstrated that PCr had an impact on blood glucose or nourishment utilization of the rats; i.e., PCr treatment diminished blood glucose level essentially as shown in Figure 2(c), compared with the diabetes group. Moreover, the body weights (BWs) of rats in the diabetic group were lower than those in the control group, while BWs were higher in diabetic rats with PCr treatment compared with the diabetes group.

#### 3.1.3. Effects of PCr on Myocardial Markers

The antioxidant activities of PCr were determined by MDA, SOD, and GSH assays using ELISA technique as shown in Figures 2(d)–2(f). These reflect the release of MDA, which was inhibited under the influence of PCr in the treated groups when compared with the STZ group, while SOD and GSH release was diminished in the STZ group when compared with the PCr-treated groups. This indicates that PCr has an antioxidant capacity.

#### 3.1.4. PCr Enhances Mitochondrial Respiration in Isolated Heart Mitochondria and Tissue Homogenate

PCr has a substrate-autonomous enhancement in the respiratory capacity as exhibited by the expansion in every respiratory parameter (state 2, OXPHOS, state 4, and electron transport system (ETS)). In fact, incitement of respiration was watched for complex I and complex II substrates, pyruvate, glutamate, malate, and succinate, individually. The integrity of the mitochondrial layer was evaluated in those examinations by including cytochrome c. In our grasp, the outcomes demonstrated that PCr enhanced ADP-animated respiration, as shown in Figures 1(c) and 1(g), by an expansion in OXPHOS for the two substrates, most presumably by filling in as an extra wellspring of electrons for the ETS similar to the control group. Additionally, PCr expanded OXPHOS in mitochondria empowered with pyruvate, glutamate, and malate. The mitochondria were stimulated with succinate, and its ETS was enhanced compared to the STZ group. A comparable inclination is seen in Figures 1(a), 1(b), 1(e), and 1(f). A comparable result was seen in the group treated with PCr (Figures 1(c) and 1(g)). Additionally, quantitative examinations of oxygen respiration rate in light of effectors are shown in Figures 1(d) and 1(h).

#### 3.1.5. PCr Modulation on a JAK2/STAT3 Signaling Pathway

**Figure 3:**
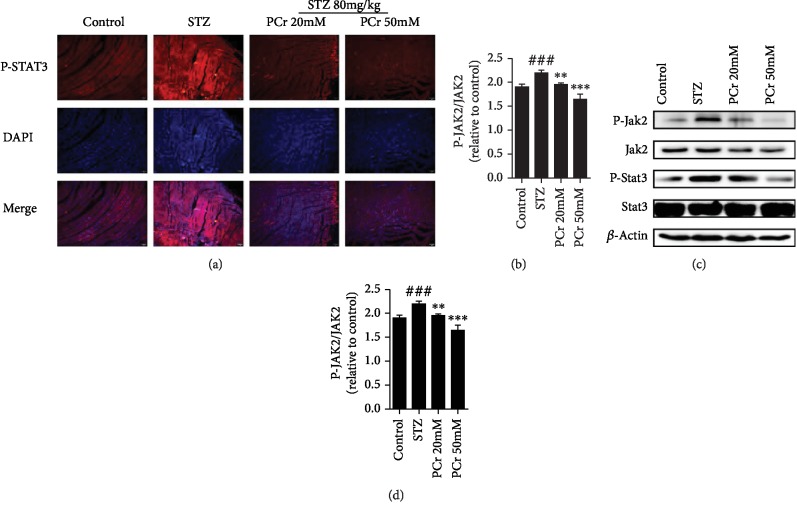
PCr modulation on the JAK2/STAT3 signaling pathway. (a) Effects of PCr on p-STAT3 translocation in ordinary and STZ conditions (original magnification 200). (b, c, d) Effects of PCr on the declaration of p-JAK2, JAK2, p-STAT3, and STAT3 in STZ and treatment as well as healthy rats (*n* = 3). Qualities are expressed as the mean ± SEM. ^##^*P* < 0.001 and ^###^*P* < 0.005*vs.* the control group. ^∗∗^*P* < 0.01 and ^∗∗∗^*P* < 0.001*vs*. the STZ group.

p-STAT3 phosphorylation in STZ assemble was expanded. PCr pretreatment specifically reversed the expanded phosphorylation of p-STAT3 in a dose-dependent manner. The immunofluorescence investigation demonstrated that p-STAT3 nuclear translocation was restrained by PCr as shown in Figure 3(a). Moreover, our results demonstrated that the protein expression of p-STAT3 was essentially expanded in a rat's myocardium after STZ infusion, while PCr treatment further diminished the declaration of p-STAT3 (Figures 3(b)–3(d)). A comparable propensity was seen in the protein expression of p-JAK. The outcomes showed that the mitochondrial pathway of apoptosis may be engaged with the pathogenesis of diabetic cardiomyopathy.

#### 3.1.6. Effects of PCr on STZ-Actuated Apoptosis in DCM

**Figure 4:**
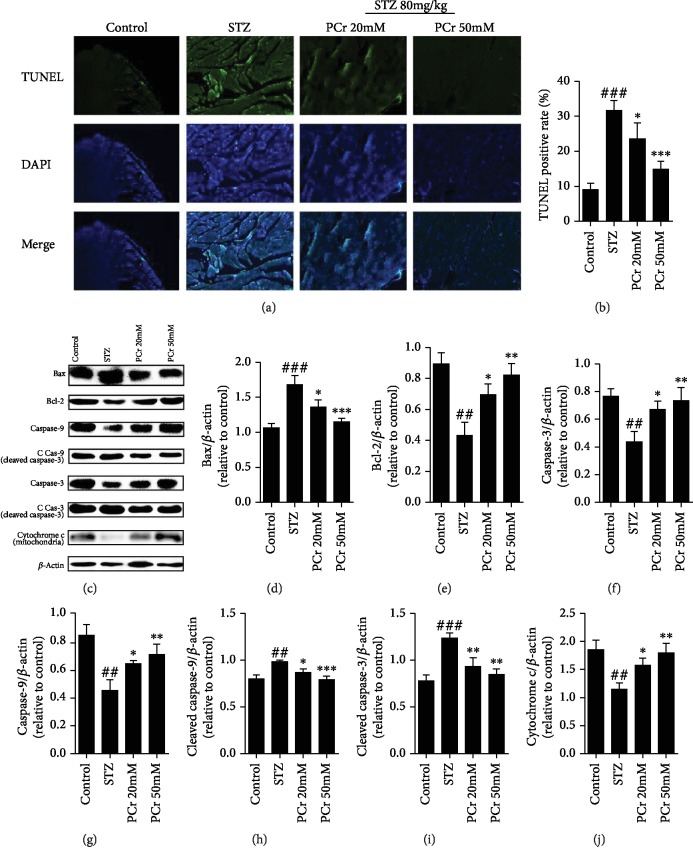
Effects of PCr on STZ-instigated apoptosis in DCM. (a) The TUNEL and DAPI recoloring on the myocardium. The nuclei of TUNEL-positive cells are shown utilizing green fluorescence. (b) Quantification of TUNEL-positive cells. (c) The protein dimensions of Bcl-2, Bax, caspase 3, caspase 9, cleaved caspase 3, cleaved caspase 9, and cytochrome c (mitochondria) were recognized by western blot in tissue homogenate. (d, e, f, g, h, i) Quantifications of western blot. Data are exhibited as the mean ± SD (*n* = 3). ^##^*P* < 0.05 and ^###^*P* < 0.01*vs*. the control group. ^∗^*P* < 0.05, ^∗∗^*P* < 0.01, and ^∗∗∗^*P* < 0.01*vs*. the STZ group.

Apoptosis actuated by STZ rats was identified utilizing TUNEL recoloring which demonstrated that STZ groups have fundamentally expanded apoptosis. Our results demonstrated that pretreatment with PCr essentially reversed the expanded apoptosis by decreasing the TUNEL-positive cells as shown in Figures 4(a) and 4(b). Furthermore, the impact of PCr on apoptosis identified with Bcl-2 family in diabetic rat hearts was examined by western blot investigation. Bcl-2 protein level was diminished, and Bax level was expanded in diabetic rats. The expression of Bcl-2 was increased, and Bax was decreased in the PCr group (Figure 4(c)). Results demonstrated that the protein expressions of caspase 3 and caspase 9 were fundamentally diminished in a rat's myocardium after STZ infusion, while being expanded after PCr treatment. Moreover, the cleaved caspase 3 and cleaved caspase 9 were altogether expanded in a rat's myocardium after STZ infusion, while being diminished after PCr treatment (Figures 4(c)–4(j)). Likewise, cytochrome c (mitochondria) was diminished in a rat's myocardium after STZ infusion while being expanded after PCr treatment. The results have shown that the mitochondrial pathway of apoptosis may be engaged with the pathogenesis of diabetic cardiomyopathy.

### 3.2. *In Vitro* Experiments

#### 3.2.1. PCr Lessened MGO-Incited Cell Damage in H9C2 Cells

**Figure 5:**
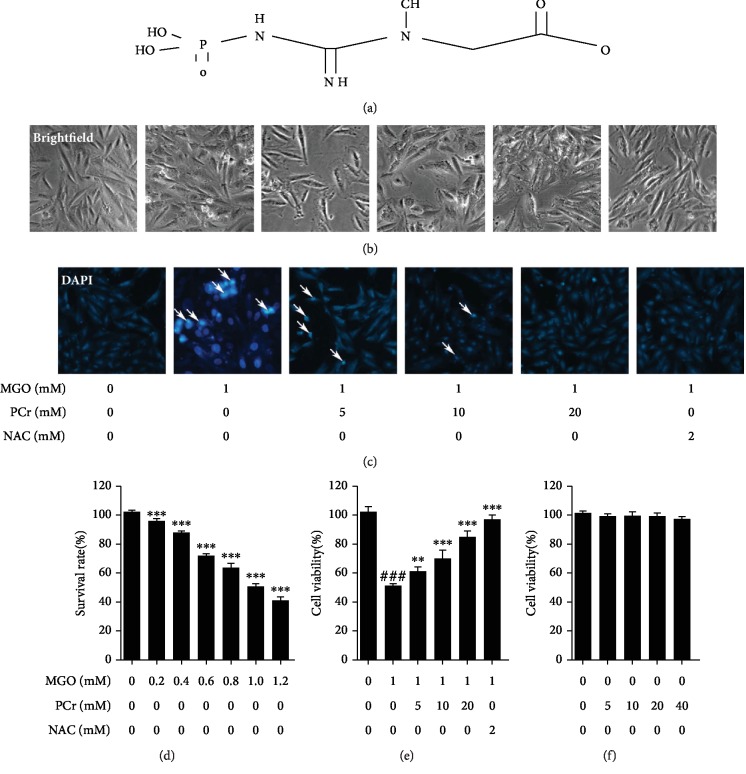
Protective impacts of PCr against MGO-actuated damage and apoptosis in H9C2 cells. (a) Chemical structure of phosphocreatine. (b) Effect of pretreatment with PCr (5~20 mM) for 2 h, on the cell morphology and structure of H9C2 cells by bright image (100x magnification) examination. (c) The apoptosis occurrences of H9C2 were stained by DAPI observed by fluorescence images for (200x final magnification). (d) MGO-induced toxicity (0.2~1.2 mM) on H9C2 cell. (e) Effect of PCr (5~20 mM) on induced MGO H9C2. (f) Cytotoxicity of PCr on H9C2 cells. The effects of PCr on the loss of cell practicality, initiated by MGO. Data are displayed as the mean ± SD (*n* = 3). ^###^*P* < 0.05*vs*. the control group. ^∗∗^*P* < 0.05 and ^∗∗∗^*P* < 0.01*vs.* the MGO group.

First of all, the chemical structure of phosphocreatine is shown in Figure 5(a). Then the cell viability test of H9C2 cell line was performed as shown in Figures 5(d)–5(f). The results indicated that PCr at different concentrations (5~40 mM) has no lethality on normal H9C2 cells. Additionally, PCr has shown protective effects on the same cell-induced injury with MGO. H9C2 cell injury was done by gradual exposure to different concentrations of MGO (0.2~1.2 mM) to induce the hyperglycemia. PCr was found to provide a significant protective effect in H9C2 cells injured by exposure to MGO.

#### 3.2.2. Improvement of Morphological Changes by PCr

As appeared in (Figure 5(b)), pretreatment with PCr for 2 h fundamentally reestablished the morphological changes of H9C2 cells including nuclear pyknosis. Through DAPI fluorescent recoloring, changes in apoptotic cells were watched. Pretreatment with PCr for 2 h portion conditionally stifled apoptosis in H9C2, as shown in (Figure 5(c)).

#### 3.2.3. PCr Inhibits MGO-Induced Apoptosis

**Figure 6:**
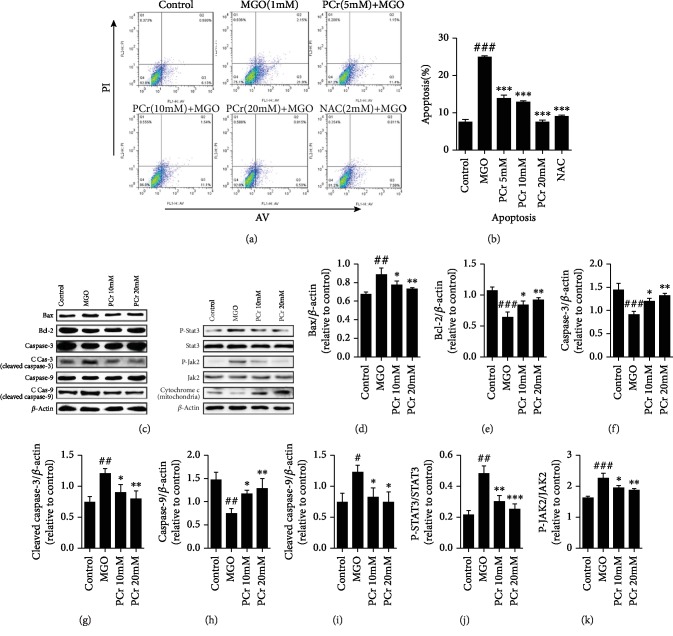
PCr inhibits MGO-induced apoptosis by modulating Bax, Bcl-2, caspase 3, caspase 9, cleaved caspase 3, cleaved caspase 9, and cytochrome c (mitochondria) in H9C2 cells. (a) Cells were treated with PCr (5~20 mM) for 2 h preceding being presented to 1 mM MGO for 24 h. Cell apoptosis was estimated by flow cytometry. (b) Apoptotic cells represent the percentage of Annexin V single positive and Annexin V/PI twofold positive cells. (c) The protein dimensions of Bcl-2, Bax, caspase 3, caspase 9, cleaved caspase 3, cleaved caspase 9, and cytochrome c (mitochondria) were recognized by western blot in cells. (d, e, f, g, h, i, j, k) Quantifications of western blot. Data are exhibited as the mean ± SD (*n* = 3). ^###^*P* < 0.05, ^##^*P* < 0.01, and ^#^*P* < 0.05*vs*. the control group. ^∗^*P* < 0.05, ^∗∗^*P* < 0.01, and ^∗∗∗^*P* < 0.05*vs*. the MGO group.

The apoptosis induced by MGO in H9C2 cells was obstructed by PCr as shown in Figures 6(a) and 6(b); it demonstrated that PCr has a defensive impact that diminished apoptosis in early and late apoptosis, showing that the concealment of apoptotic cells was diminished by PCr in a dose-dependent manner. This impact had been researched with Annexin V-FITC and PI double recoloring and was performed by utilizing flow cytometry investigation.

#### 3.2.4. Effects of PCr on the Expression of Proteins Associated with DCM

The impacts of PCr on apoptosis-related Bcl-2 family and the JAK2/STAT3 pathway actuated by MGO were studied by western blot investigation. Bcl-2 protein level was diminished, and Bax level was expanded in the MGO group, bringing about a higher distinction, contrasting in the treatment groups. Additionally, cytochrome c (mitochondria) was diminished in the MGO group while being expanded after PCr treatment. The outcomes have shown that the mitochondrial pathway of apoptosis may be associated with the pathogenesis of DCM. PCr treatment fundamentally expanded Bcl-2 expression and diminished Bax expression (Figures 6(c)–6(i)). Results demonstrated that the protein expression of p-JAK was essentially expanded in the MGO-instigated group, while PCr treatment further diminished the outflow of p-JAK. A comparable propensity was seen in the protein expression of p-STAT3 (Figures 6(c), 6(j), and 6(k)).

#### 3.2.5. PCr Improves Mitochondrial Respiration

**Figure 7:**
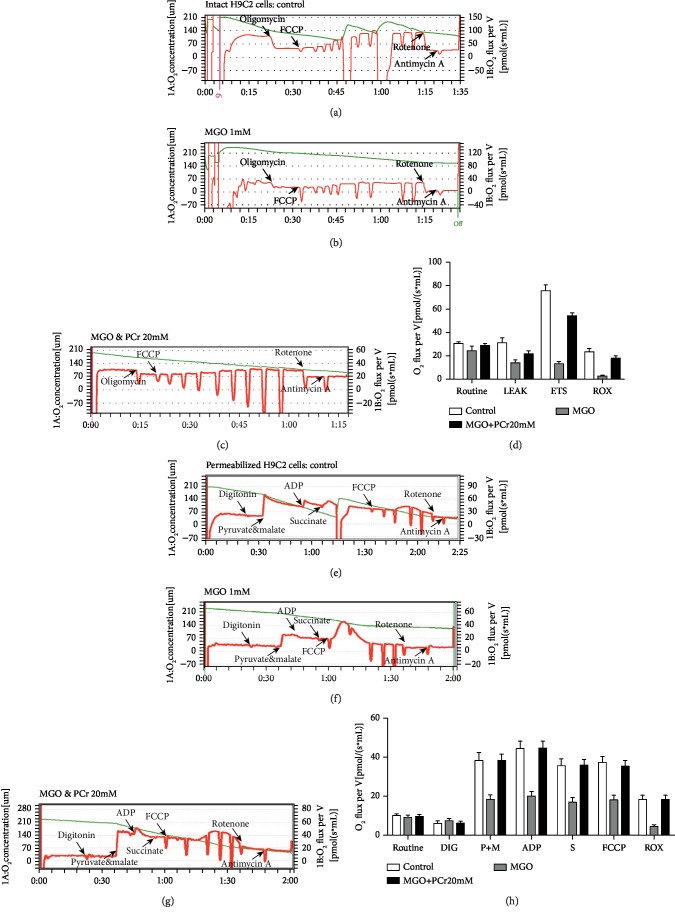
PCr enhances mitochondrial respiration. (a) Effects of PCr on mitochondrial respiration in intact H9C2 cells. Agent respiratory explore different avenues regarding control H9C2 cells. (b) MGO-incited H9C2 cells. (c) PCr (20 mM) pretreated with MGO (1 mM) invigorated cells. (d) Quantification for all groups in intact H9C2 cells. The respirometry convention for intact cells included the ROUTINE state which was estimated when a steady oxygen transition had been gotten following expansion of cells into the test chamber. Uncoupled respiration in the LEAK state was estimated when a consistent oxygen motion had been accomplished after the expansion of oligomycin (2.5 *μ*M), FCCP (0.5 *μ*M steps), rotenone (0.5 *μ*M), and antimycin A (2.5 *μ*M). (e) Effect of PCr on permeabilized H9C2 cells in control. (f) MGO-induced H9C2 cells. (g) PCr (20 mM) pretreated with MGO-stimulated cells. (h) Quantification for all the groups in permeabilized H9C2 cells. The following are added: digitonin (8.1 *μ*M, 10 *μ*g/10^6^ cells); P: pyruvate (5 mM); G: glutamate (10 mM); M: malate (2 mM); ADP (5 mM); S: succinate (10 mM); FCCP (0.5 *μ*M steps); rotenone (0.5 *μ*M); antimycin A (2.5 *μ*M). Data are presented as the mean ± SD (*n* = 3).

In an ordinary cell culture, mitochondrion gives the greater part of the vitality produced under typical conditions. The generation of energy in mitochondria can be estimated by mitochondrial oxidative phosphorylation limit (oxygen flux or oxygen consumption rate (OCR)) and oxygen concentration (Figures 7(a) and 7(e)). OCR was assessed utilizing high-goal respirometry. After a standard OCR was recorded, oligomycin was included and the oligomycin safe respiration rate or nonphosphorylating respiration was resolved. Maximal respiratory capacity (MRC) alludes to the most extreme animated respiration of the electron transport chain (ETC) (complex I~V incorporated movement) by FCCP. The control group had higher OCR of basal, hole, and FCCP than the MGO-actuated damage group as shown in Figures 7(b) and 7(f). Nevertheless, the PCr pretreatment had higher OCR essentially extraordinary in contrast with the MGO group. Also, the coupling proficiency was like the control (Figures 7(a) and 7(c)). The rate of oligomycin-safe respiration is frequently optional to proton leak, while in the ATP blend, proton leak also impacts substrate oxidation on the baseline OCR. Undoubtedly, our result indicated that oligomycin-safe OCR in the MGO group was significantly lower compared with that in the control group and the PCr pretreatment group showed significantly increased OCR compared with the MGO group. It was recommended that there was an expansion in ATP combination by complex V in these cells. Recommending there was an expansion in ATP combination by complex V in these cells. Besides, the proof in help of expanded mitochondrial capacity can be found in the reality the save control group and PCr group likewise had higher ETC than MGO amass recommending that MGO group capacity closer to their bioenergetics potential at instrument when looked with expanding needs was not ready to enlarge vitality creation. Overall, this information recommends that PCr demonstrations fortify mitochondrial and oxidative limit. Likewise, in permeabilization of H9C2 cells, PCr possessed a substrate-autonomous enhancement in the respiratory capacity as shown by the expansion in every single respiratory parameter (state 2, OXPHOS, state 4, and ETS) in both control and treated groups with PCr (Figures 7(e) and 7(g)). The incitement of respiration was observed for complex I and complex II substrates, i.e., pyruvate, glutamate, malate, and succinate, separately. PCr enhanced ADP-animated respiration, as shown by an expansion in OXPHOS for the two substrates, most likely by filling in as an extra wellspring of electrons for the ETS. Similar to the control group, PCr expanded OXPHOS in mitochondria empowered with pyruvate, glutamate, and malate. The mitochondria were invigorated with succinate contrasted with the MGO-actuated group. Also, a comparable propensity was seen in both intact and permeabilized H9C2 cells as shown in Figures 7(d) and 7(h).

#### 3.2.6. Improvement of Mitochondrial Membrane Permeability (*ΔΨ*m) by PCr on the MGO-Harmed H9C2 Cells

**Figure 8:**
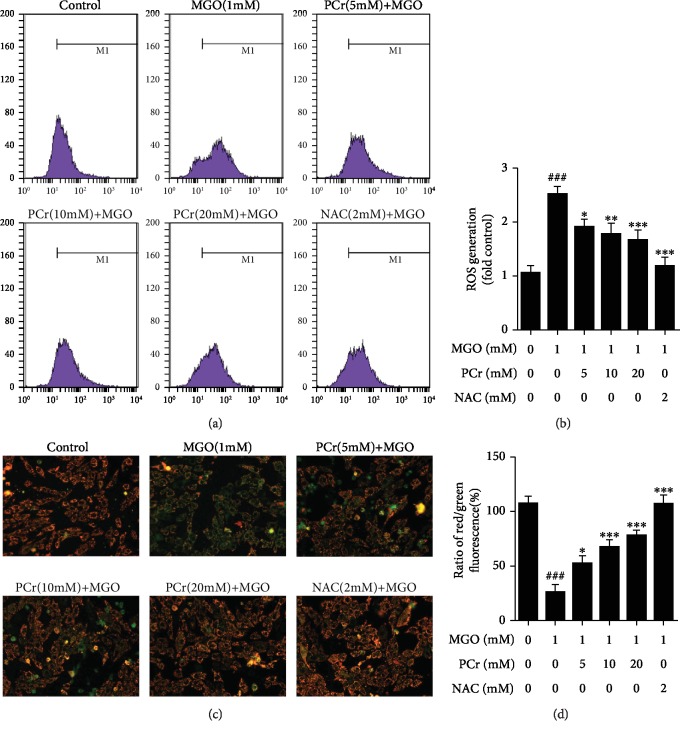
Inhibitory impacts of PCr on MGO-actuated H9C2 ROS overproduction. (a) Cells were treated with convergences of (5~20 mM) PCr or 2 mM NAC for 2 h before being animated with (1 mM) MGO for 24 h. (b) Quantification ROS age. (c) Protective impacts of PCr against MGO-initiated mitochondrial brokenness in H9C2 cells. The impact of PCr on mitochondrial membrane potential. The *Δψ*m in each group was computed as the proportion of red to green fluorescence. (d) Data are presented as the mean ± SD (*n* = 3). ^###^*P* < 0.05*vs*. the control group. ^∗^*P* < 0.05, ^∗∗^*P* < 0.01, and ^∗∗∗^*P* < 0.01*vs*. the MGO group.

We assessed MMP utilizing the JC-1 test to test the counter apoptotic impacts of PCr (Figures 8(c) and 8(d)). Suitable cells were shown with red fluorescence, implying a high MMP while apoptotic cells show green fluorescence implying a low MMP (*Δψ*m). After the H9C2 cells applied with MGO for 24 h, MMP was depolarized in MGO-treated cells as shown by the increase in green fluorescence, while pretreatment of PCr kept the condition in *Δψ*m as shown by the decrease in red fluorescence.

#### 3.2.7. Suppression of Intracellular ROS Generation

To analyze whether the expanded oxidative pressure is related to MGO-prompted apoptosis in H9C2, flow cytometry examination by DCFH-DA recoloring was affirmed (Figures 8(a) and 8(b)). The H9C2 cells with MGO (1 mM) for 24 h notably caused ROS generation compared with control, while treatment with PCr for 2 h conditionally smothered ROS creation in the H9C2 cells.

#### 3.2.8. Modulation of PCr on the p-STAT3 Pathway in H9C2 Cells

**Figure 9:**
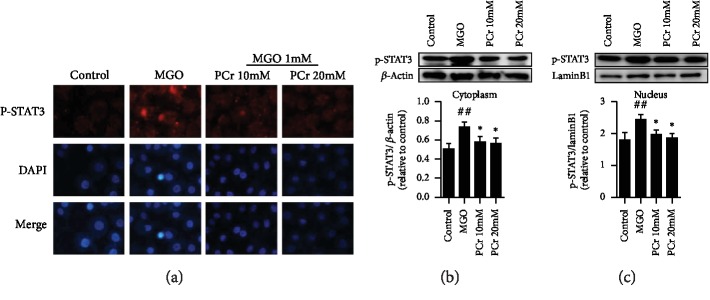
Effects of PCr on the expression level of pSTAT signaling pathway. (a) Immunofluorescence recoloring examination of pSTAT confinement. The H9C2 cells were named with pSTAT (red), and the nuclei were recolored with DAPI (blue). (b, c, d) Western blot examination of pSTAT in the cytoplasm and nucleus. Data are introduced as the mean ± SD (*n* = 3). ^∗^*P* < 0.05 and ^∗∗^*P* < 0.01 significantly different from the control group.

We have analyzed the phosphorylated and aggregate expression dimension of p-STAT3, after being treated with PCr in H9C2 cells initiated by MGO using immunofluorescence recoloring and western blot. As shown in Figure 9(a), the pretreatment with PCr (10 and 20 mM) for 2 h essentially diminished the nuclear translocation of p-STAT3. In Figure 9(a), p-STAT3 was dominatingly situated in the cytoplasm of H9C2 cells in the model group. What is more, the fluorescence force of the nuclear p-STAT3 was diminished essentially in a dose-dependet manner after PCr treatment individually; in contrast with the model group, as shown in Figure 9(a), pretreatment of PCr (20 mM) clearly is reliable with the outcome that the nuclear p-STAT3 levels were diminished in the cytoplasm by western blot test as shown in Figures 9(b) and 9(c).

## 4. Discussion

DCM, one of the most severe cardiovascular complications, can cause cardiac dysfunction in diabetic patients [29]. DCM is characterized with cardiac functional and structural changes, such as cardiac hypertrophy, oxidative stress, apoptosis, and myocardial interstitial fibrosis, which are the principal features of DCM [1, 30, 31]. Our histopathological study has shown markedly structural changes such as abnormal striation in myocardium tissue in the untreated group compared with the treated group, with a clear improvement of the myocardium striation as well as a reduction of heart size in treated groups, indicating that PCr is a novel therapeutic choice against the major features of DCM, while the morphology of H9C2 has also been improved in the treated group rather than the MGO-induced group.

Treatment with PCr (20~50 mM) in rats shows that PCr effectively reduced the blood glucose level and improved diabetes symptoms slightly, suggesting that the reduction of blood glucose level by PCr may be one of the mechanisms of improving heart morphology and function in diabetes.

Impressive proof proposes that overproduction of ROS actuated by hyperglycemia is an unequivocal factor in the improvement of DCM [32, 33]. Ongoing investigations have recommended that hyperglycemia-induced oxidative damage plays an important role in the early stage of DCM [34]. Our present study showed that PCr could create a protective effect against ROS as well as MGO-induced H9C2 cell injury by antioxidant activities, which was consistent with our previous study regarding the antioxidant activities of PCr in MGO-induced endothelial cells [35, 36].

**Figure 10:**
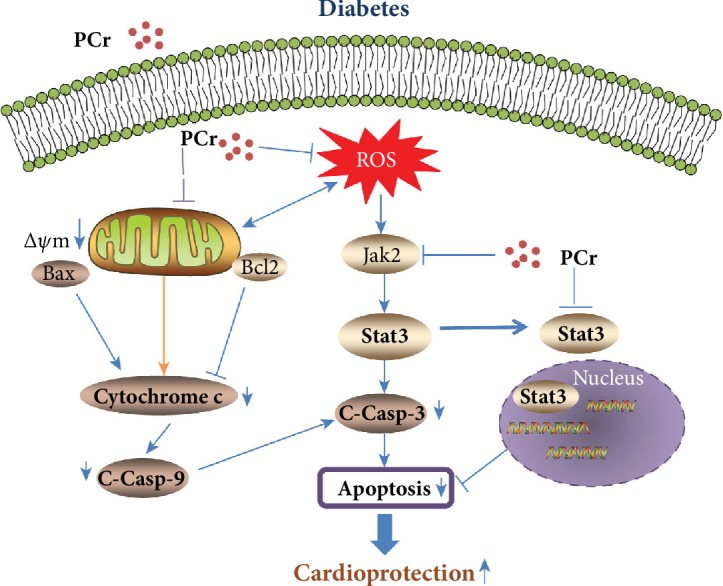
Schematic representation influence of PCr against DCM on Bcl-2 family members and other mitochondrial apoptotic and metabolic proteins in cardiomyocytes under various pathological conditions.

Augmented myocardial cell apoptosis is an imperative occasion in the improvement of DCM [4]. Recent studies have described the role of STAT3 in apoptosis, demonstrating that inhibition of STAT3 suppresses cleaved caspase 3 [43]. In our present examination, the after effects in vivo and in vitro demonstrated that PCr likewise bolstered the report, i.e., the inhibition of STAT3 stifled caspase-3. In addition, we found that PCr could improve the cardiomyopathy by repressing oxidative pressure and balancing the mitochondrial pathway through the decreased apoptosis pathway. These discoveries demonstrated that PCr may be a plausibility in the improvement of diabetic cardiomyopathy. Our study proposes new aspects to the signal transduction pathway of this PCr-mediated protection and emphasized the involvement of mitochondrial signaling pathways (Figure 10).

Moreover, SOD, MDA, and GSH are enzymatic prevention agents that assume a fundamental job in keeping cells from being presented to oxidative harm in diabetes mellitus [37]. Our results showed that significant impacts of PCr on SOD, MDA, and GSH reveal potent antioxidant activity. In addition, an in vitro study showed that ROS was markedly increased in the MGO group compared with the treated group in different concentrations of PCr in a dose-dependent manner. The rising proof proposes that DCM is connected to adjustments in myocardial fuel and energy metabolism. Diverse trial reports have shown that the mitochondria assume an essential job in the pathogenesis of diabetes [38]. It is known that diabetic cardiomyopathy (DCM) is involved in glucose and lipid metabolism disorder, oxidative stress, inflammation, apoptosis, and so on. Our results showed that mitochondrial dysfunction was closely related to multiple pathogenic links of DCM. Mitochondria are important sites of energy metabolism in cells, compared with other muscle cells. The heart is rich in mitochondria. The activity of mitochondrial respiratory chain-related enzymes in rats was significantly decreased [44]. Type 2 diabetes' mitochondrial respiratory function was impaired in ob/ob and db/db mice [45, 46]. We are sure that our clinical advances will enlighten clinical doctors to explore effective prevention and treatment measures targeting the mitochondria. In addition, mitochondrial brokenness is for all intents and purposes at the center of every single cardiovascular issue and associated with the maturing procedure. Besides, it has been accounted for that keeping mitochondria in a solid state is a confounded procedure and must be firmly managed by means of mitochondrial quality control instruments and complex transaction between mitochondrial biogenesis and degradation [39]. Despite the mitochondria being the primary generator of ROS, they are likewise helpless to the harming impact of ROS. In the mix, the changes in diabetes-actuated mitochondria are very much depicted; the changes in the primary parameters of mitochondrial respiration would thus prompt the confinement of ATP generation and most likely the expansion in the ROS arrangement. In this manner, the capacity of mitochondria is closely linked to the maintenance of redox [40]. Our results indicated that the application of the measurement of mitochondrial respiration could be a potential sensitive assay for cellular dysfunction from STZ and MGO poisoning. These recommended that decreased effectiveness of mitochondrial respiration by PCr has been exhibited in diabetes, especially for the exceptional need in tissues. Our results additionally showed that this recuperation of mitochondrial respiration by PCr diminished unsettling influences of mitochondrial works because of the increase of the electron transport chain action and ATP creation. It is shown that the estimation of mitochondrial respiration may hold more noteworthy utility in this regard [41]. In the current study, we further found that the mitochondrial respiration function in the isolated heart mitochondria or heart tissue homogenated groups treated with PCr-induced STZ could be elevated to recover the same with the control group, showing a normal response similar to the control group, which means they expired from STZ poisoning. We also found that ETS, which represents the mitochondrial bioenergetics reserve, was significantly decreased in the STZ group. For the supporting evidence of this notion in both tissue homogenated and isolated heart mitochondria, we observed that the rats treated with the highest PCr dose (50 mM/day) displayed a huge propensity to have the most astounding substrate affectability in contrast with the STZ group and control subject. Moreover, the past examination has demonstrated that the depolarization in mitochondrial film potential (MMP) is an element of apoptosis. Intracellular ROS creation has been shown to prompt apoptosis by uncontrollable MMP [42]. Exorbitant intracellular ROS creation has been shown to prompt apoptosis by boisterous MMP. Our outcome affirmed that the counter apoptotic activity of PCr was intervened by the concealment of mitochondrial layer potential condition in order to hinder the mitochondrial apoptotic pathway and to additionally forestall DCM. Moreover, our results demonstrated that the apoptosis pathway significantly reduced as depicted in flow cytometry as well as TUNEL assay. Moreover, PCr possesses the antiapoptotic effect *in vivo* and *in vitro* when using western blot in changing the protein expression of apoptotic proteins such as Bcl-2, which was decreased in the model group and increased in the treatment groups compared with the control group. In addition, Bax was expanded in the model group and diminished in the treatment group in contrast with the control group. Furthermore, caspase 3 and caspase 9 were diminished in the model group and expanded in the treatment group, and also cleaved caspase 3 and cleaved caspase 9 were expanded in the model group and diminished in the treatment group with PCr. Moreover, the nuclear translocation of p-STAT3 was detected to be higher in the STZ group than that of the PCr group, reflecting the improvement of PCr against DCM.

## 5. Conclusion

The simultaneous measurement of respiration greatly enhances the informative potential of studies of mitochondria. Our outcomes are given proof that PCr can avoid hyperglycemia-initiated myocardial oxidative pressure, mitochondrial brokenness, and protected cardiovascular dysfunction. The pretreatment with PCr is an effective protective agent against the complications associated with diabetes in H9C2 and in cardiac tissue from rats, being treated with STZ to induce experimental diabetes. More specifically, pretreatment with PCr has been found to arrest apoptosis triggered by hyperglycemia. Although, PCr preserved the normal morphology of cardiac cells exposed to MGO through the restraint of the JAK2/STAT3 signaling pathway. Besides, PCr may fill in as a novel restorative methodology for enhancing and balancing out mitochondrial work and a defensive impact against DCM.

## Data Availability

All data are preserved by Eskandar Qaed and Zeyao Tang.

## Abbreviations

ROS: Reactive oxygen species
JAK/STAT: Janus kinase/signal transducer and activator of transcription.
